# Supplemented Very Low Protein Diet (sVLPD) in Patients with Advanced Chronic Renal Failure: Clinical and Economic Benefits

**DOI:** 10.3390/nu15163568

**Published:** 2023-08-13

**Authors:** Sofia Cecchi, Silvio Di Stante, Sara Belcastro, Veronica Bertuzzi, Assunta Cardillo, Laura Diotallevi, Xhensila Grabocka, Hrissa Kulurianu, Mauro Martello, Valentina Nastasi, Osmy Paci Della Costanza, Francesca Pizzolante, Marina Di Luca

**Affiliations:** 1 Department of Clinical and Molecular Science, Polytechnic University of Marche, 60121 Ancona, Italy; 2 Department of Nephrology and Dialysis, Ospedale Santa Croce, Azienda Sanitaria Territoriale n 1, Pesaro-Urbino, 61032 Fano, Italymarina.diluca@ospedalimarchenord.it (M.D.L.)

**Keywords:** low protein diet, renal nutrition, chronic kidney disease, ketoanalogues, amino acid, dialysis, malnutrition, cost benefits

## Abstract

The supplemented very low-protein diet (sVLPD) has proven effective in slowing the progression of stage 5 chronic renal failure and postponing the start of the dialysis treatment. However, sVLPD could expose the patient to the risk of malnutrition. This diet is also difficult to implement due to the required intake of large number of keto-analogue/amino acid tablets. In our Center, the Department of Nephrology and Dialysis of Azienda Sanitaria Territoriale n 1, Pesaro-Urbino, of Italy, respecting the guidelines of normal clinical practice, we prescribed sVLPD (0.3 g/prot/day) supplemented with only essential amino acids without the use of ketoanalogues in stage 5 patients and verified its efficacy, safety and clinical and economic effects. Over the 24 months period of observation the progression of chronic kidney disease (CKD) slowed down (mean eGFR 11.6 ± 3.3 vs. 9.3 ± 2.7 mL/min/1.73 m^2^, *p* < 0.001) and the start of the dialysis treatment (adjusted HR = 0.361, CI 0.200–0.650, *p* = 0.001) was delayed without evidence of malnutrition, in compliant vs. non-compliant patients. This led to a substantial cost reduction for the National Health System. This non-interventional longitudinal observational study is part of standard clinical practice and suggests that VLPD supplemented with essential amino acids could be extensively used to reduce the incidence of dialysis treatments, with a favorable economic impact on the NHS.

## 1. Introduction

Chronic renal failure [1] has a negative impact on patients’ morbidity and mortality as well as on healthcare costs [2,3,4]. The number of patients with end-stage chronic renal disease (ESRD) is constantly increasing [5] and so are the projected costs to be sustained by the Health Services [1]. It is also known that dialysis compared to conservative therapy does not improve the quality of life nor it prolongs survival in specific patient populations [6].

Diet is one of the main modifiable risk factors for CKD [7] and reducing protein intake is considered an effective and safe tool in the conservative treatment of renal disease [8,9,10,11,12,13], particularly in the CKD stage 5 [4,13,14,15,16,17,18].

Dietary-nutritional treatment (DNT) should be regarded as a proper therapy and it should be integrated with the pharmacological therapy [16,19,20], with the aim of increasing life expectancy by reducing the progression of renal damage and postponing the start of dialysis [8,13,14,21,22,23,24]. Furthermore, DNT allows a patient to achieve good metabolic control, prevent and correct uremic symptoms and metabolic acidosis, and to rebalance electrolytes, especially potassium, phosphorus and sodium [1,3,9,12,16,19,25].

Although to date, there are conflicting opinions on the use and benefits of DNT in CKD [1,26,27,28] and the investigation is ongoing, the latest KDOQI 2020 guidelines recommend a strict hypoproteic diet in stages 3–5 and several studies show that the use of sVLPD in patients with advanced chronic renal failure yields greater benefits than a low-protein diet (LPD) alone [11,12,23,29,30,31]. It is not clear whether the effects emerging from the use of sVLPD with KAs/eAAs are attributable to the reduced protein intake or to the role of KAs, in the short and long term, in stage 5 patients [15,32,33,34].

One of the main controversial aspects is that sVLPD can induce malnutrition with protein-energy depletion (PEW) [1,3,12,16,35,36]; however, this can be overcome through adequate caloric intake [26] and patient monitoring [8,12,13,37,38]. Metabolic acidosis that comes from excess dietary protein increases muscle catabolism and the risk of PEW. Therefore, the correction of acidosis through a low-protein diet contributes to reducing the acid load and consequently to preserve muscle mass and to slow down the progression of renal damage [39,40,41,42].

sVLPD is not widely adopted also due to the reduced compliance [1,23,27,43,44,45,46,47,48,49] by patients who are required take a large number of ketoanalogue tablets [15,50,51]. It is also known that if dietary adherence is low, prescribing sVLPD does not provide additional benefits over standard LPD [1]. Some authors have analyzed this aspect concluding that adherence to the diet is not predictable beforehand and have indicated strategies to improve compliance, such as nutritional education, careful patient selection, prescription of personalized diets and regular follow-up visits [48].

Previous studies, both simulation and observational, have shown that adopting low-protein diets contributes to cutting costs by postponing the beginning of dialysis treatment [50,52,53], and that the benefits are more substantial the earlier the diet is started [4,10,19,50,52,53].

In the Italian National Chronicity Plan (PNC) published in September 2016, DNT was indicated as an “integral part of the conservative treatment of CRF” and its adoption was promoted in an “individualized way to the maximum possible degree … (omissis) … gradually and progressive taking into account the stage of the disease”.

In a recent position statement of the Italian Society of Nephrology concerning the strategies to reduce the environmental impact of dialysis, diet is indicated as one of the useful tools for this purpose, delaying the start of replacement treatment [54].

In our center, we opted to prescribe sVLPD (0.3 g/kg/day) supplemented only with essential amino acids without the use of ketoanalogues in patients with eGFR <15 mL/min/1.73 m^2^.

The aim of this retrospective observational study is to evaluate the safety and efficacy of sVLPD with AA in slowing down the progression of CKD and delaying the beginning of dialysis treatment with consequent cost savings. A further objective was to verify whether patient compliance was actually higher with this diet therapy approach compared to sVLPD with ketoanalogues, thanks to the reduction in the pill burden.

We analyzed nitrogen metabolism, electrolyte and acid-base balance, iron balance, body composition using bioimpedancemetry, differences between compliant vs. non-compliant patients, and mean eGFR reduction before and after sVLPD inception.

## 2. Materials and Methods

A total of 129 adult patients referred to the advanced renal disease ambulatory were started on sVLPD supplemented with essential amino acids and individually customized by a dedicated dietitian. A total of 101 patients had already been prescribed LPD in CKD stages 3 and 4 (Table 1).

Patients were evaluated monthly, based on eGFR and clinical stability, with blood tests (BUN, creatinine, eGFR calculated with the MDRD equation, uric acid, glycemia, calcium, phosphorus, potassium, sodium, chloremia, magnesium, albuminemia, blood gas analysis) and vital signs. Anthropometric measurements (weight, BMI) and body composition (intra and extracellular water, lean mass, fat mass) were measured quarterly using Bioelectrical Impedance Analysis (BCM Fresenius Medical Care) and protein intake, calculated and based on azoturia/day (6.25 × [UUN g/24 + (30/Weight kg)]/100), including the amount derived from amino acid supplementation, estimated at about 0.1–0.2 g/kg/day.

Monthly checks were carried out with the dietician both in presence and remotely. During the observation, iron, folic acid, erythropoietin, group B and D vitamins, phosphorus and potassium chelators, and bicarbonate supplements were prescribed according to the laboratory findings and the clinical assessment.

### 2.1. Features of the Diet

The diet featured a caloric intake of 25 Kcal/kg/day [26], protein intake of 0.3 g/prot/day with amino acid supplementation (composition and dosage of amino acids used are shown in Table 2), carbohydrate intake derived mainly from protein-free products and fruit, sodium intake ≤1.5 g/day [55], P intake 300–400 mg/day [6,20,56] and potassium intake 2000 mg/day.

The indication to start the dialysis treatment was based on the following clinical evaluations: hyperazotemia, eGFR ≤ 6 mL/min/1.73 m^2^, hyperkalemia, poor blood pressure control, uremic symptoms (gastrointestinal disturbances, asthenia, restless legs syndrome), hypervolemia, and metabolic acidosis.

### 2.2. Inclusion Criteria

Adult patients referred to the advanced renal disease ambulatory with eGFR (MDRD) equal to or lower than 15 mL/min/1.73 m^2^, with at least 3 months of observation.

### 2.3. Exclusion Criteria

- Patient refusal;
- Objective impossibility of the patient (e.g., socio-economic or psychological discomfort);
- Cachectic patient and/or BMI <18.5 kg/m^2^ and/or albumin <3 g/dL;
- Intolerance to protein-free products and/or essential amino acids;
- Patients with acute pathological conditions, neoplastic, pregnant or breastfeeding, with very reduced life expectancy or other acute or chronic catabolic states;
- Patients with pathological conditions treated with cytotoxic drugs and/or steroids;
- Patients on enteral/parenteral nutrition;
- Eating behavior disorders;
- Decompensated diabetes;
- Chronic inflammatory bowel disease or other conditions potentially causing malabsorption.

### 2.4. Definition of Non-Compliant Patient (with at Least One Other Criterion in Addition to the First)

**Figure 1:**
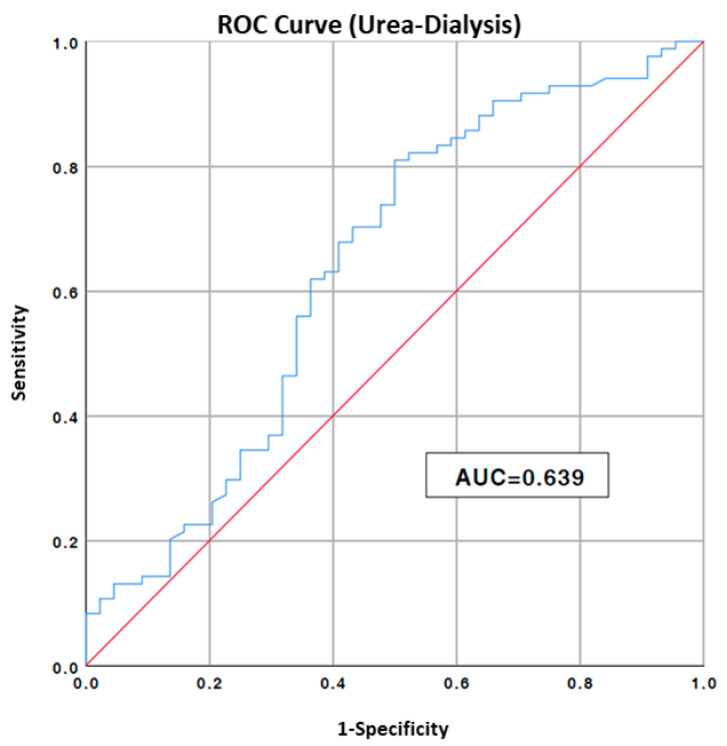
ROC curve to estimate urea cut-off value to risk of hemodialysis; AUC, area under the curve. Blue line: values of our data, Red line: no predictive value.

- Non-compliance declared by patient/caregiver;
- Mean blood urea nitrogen > 128 mg/dL, cut-off value identified with the ROC curve for dialysis event (Figure 1);

- Persistently measured protein intake >0.5 g/kg/day

All patients gave their consent to the nutritional therapy which was proposed following normal clinical practice, and everything was conducted according to the guidelines of the Declaration of Helsinki.

### 2.5. Statistical and Economic Analyses

The descriptive analysis was performed as mean ± standard deviation or median ± interquartile. Categorical variables were reported as absolute numbers and percentages.

Student’s *t* test was used to compare the means of the individual parameters in the total sample examined and between compliant and non-compliant patients. The Chi-square test or Fisher’s exact test was used to compare categorical variables and to analyze the changes in body composition. The Kaplan–Meier curves were used to compare the cumulative incidence of events between subpopulations and the log-rank calculation was used to assess the statistical significance. The analysis of the risk (HR) of ESRD, mortality and renal death with 95% CI was calculated with the Cox linear regression model. Statistical significance was considered for *p* < 0.05.

The body composition by three parameters (total water, lean mass, fat mass) was measured using bioelectrical impedance analysis. Since the instrument reference ranges vary according to a patient’s BMI, the means of the absolute values measured were not comparable. Therefore, the percentages of normal range patients were considered for the comparison between the two groups.

For the evaluation of the economic savings in terms of months of dialysis avoided, the following criteria were assumed: eGFR, at which the patients would have started the replacement treatment if they had not followed a sVLPD equal to 10 mL/min/1.73 m^2^, an annual loss estimated average eGFR of 3 mL/min/1.73 m^2^, average cost of dialysis treatments 40,000 EUR /year/patient (Adapted HTA REPORT*: “HTA Evaluation of Dialysis Methods in Italy” 2015 Ministry of Health).

Software used: SPSS version 26.0.

## 3. Results

As reported in Table 1, at the end of the 24 months of observation, the data of 129 patients with at least 3 months of follow up were analyzed. The average age was 75.2 years, 60.5% were male, all hypertensive, 40.3% were diabetic and 24.8% had heart disease. A proportion of 78.3% (101 pts) were previously prescribed a low-protein diet (LPD) (0.8 to 0.6 g/kg/day).

Table 3 shows the age, the anthropometric parameters, the BP and the values of the blood chemistry tests at the beginning of the observation of the three groups (whole population, compliant and non-compliant patients); at baseline non-compliant patients had a higher weight, BMI and creatinine than compliant patients, but not a statistically significant lower eGFR. There were no differences between the two groups for all other measured parameters.

During the observation, there were 24 deaths (13 pts over 85 years old), 20 peritoneal dialysis admissions, 36 hemodialysis admissions, 4 pre-emptive living transplants and 15 renal functional recoveries with eGFR consistently >15 mL/min/1.73 m^2^ (Table 4).

The mean observation time was 13.6 ± 6.8 months for a total of 1759.5 months of diet (Table 4). During the observation, 91 patients (70.5%) were compliant with the dietary therapy, based on the criteria listed above, a much higher percentage than reported in previous studies (15–26%).

The comparison of the mean values of the parameters monitored in the two groups during the observation is summarized in Table 5.

**Figure 2:**
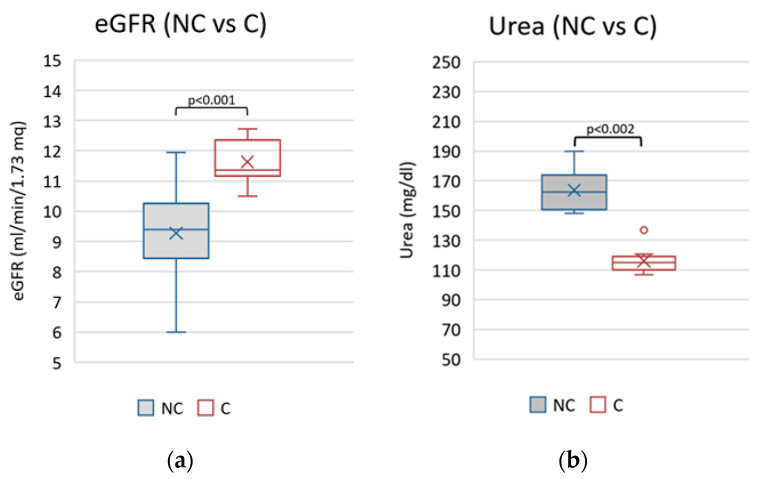
Comparison between mean blood urea nitrogen (**a**) and eGFR (**b**) values between compliant and non-compliant patients. NC, non-compliants; C, compliants.

By comparing the mean parameters of the two groups, statistically significant differences were observed for blood urea nitrogen and eGFR (Figure 2).

**Figure 3:**
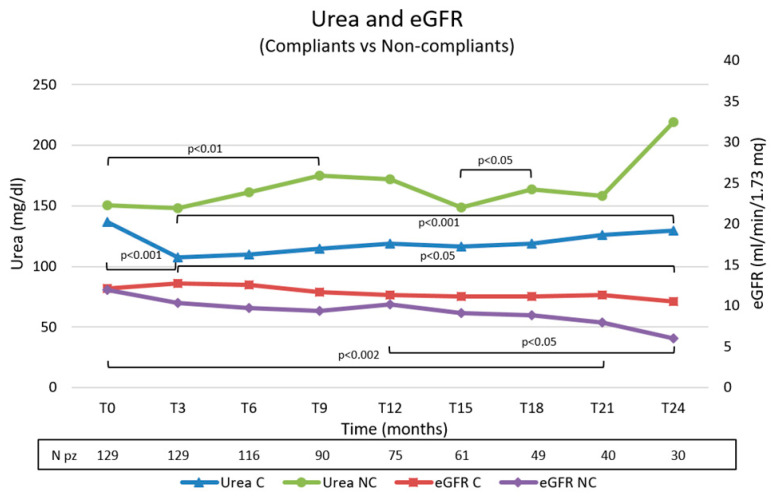
Trend of urea and eGFR values in the compliant and non-compliant group during the observation. C, compliants; NC, non-compliants.

The trend during the observation of the two parameters is shown in Figure 3. We observed significative better control of urea values and a minor decrease in eGFR in the compliant group.

The differences in weight and BMI between the two groups at the beginning of the observation were maintained and significantly accentuated throughout the observation period, with an average BMI value higher than 30 kg/mq (first degree obesity) in the non-compliant group (Table 5).

With regard to the body composition parameters (total water, lean and fat mass) the mean percentage of in-range patients was higher in the compliant group, although not statistically significant (Table 5).

In the compliant group, there was an improvement trend both for lean mass and for fat mass, without signs of malnutrition (Supplementary Figure S1).

**Figure 4:**
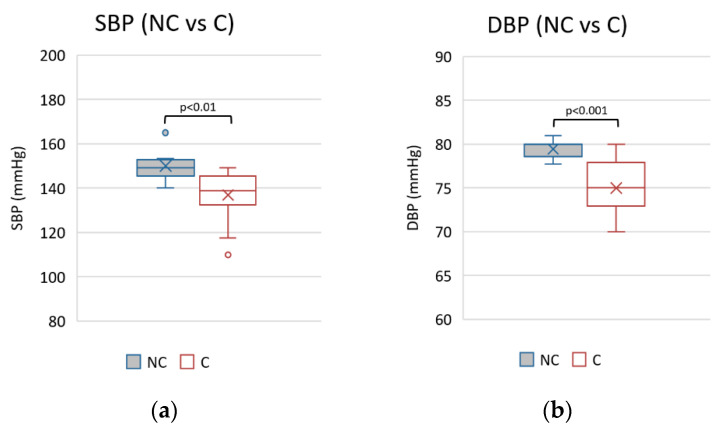
Mean values of systolic (**a**) and diastolic (**b**) blood pressure during observational period in non-compliants (NC) vs. compliants (C).

Better pressure control was observed in the mean values of SBP and DBP in the compliant group (Figure 4).

The mean TSAT values were higher in the compliant group despite a higher mean transferrin levels in the non-compliant (Table 5).

Although the metabolic acidosis was constantly well compensated in both groups with mean bicarbonate values within the normal range (nv 22–29), there was a significant improving trend in the bicarbonate level in the compliant group and a worsening trend in the non-compliant group over time (Supplementary Figure S2).

**Figure 5:**
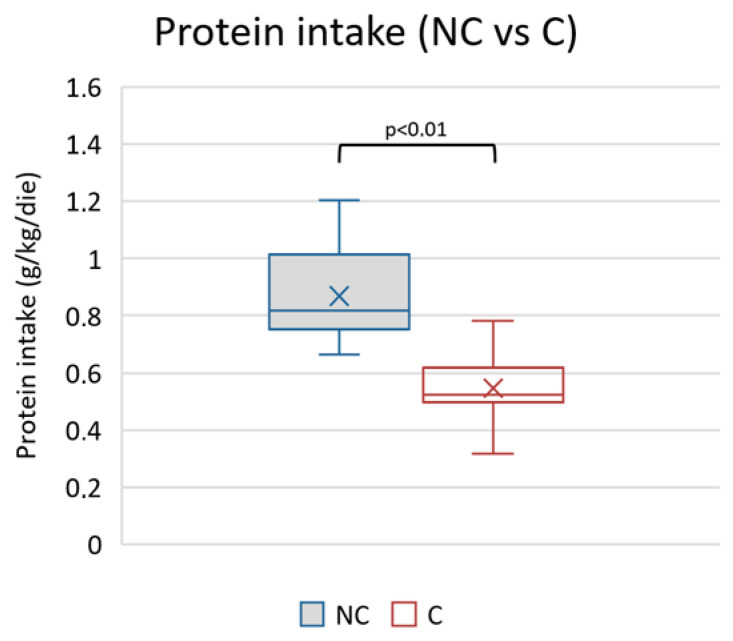
Mean values of protein intake during observational period in non-compliants (NC) vs. compliants (C).

The average protein intake in the observed period was different between the two groups (higher in the non-compliant) (Table 5 and Figure 5).

On average, uricemia was always within the normal range in both groups but was significantly lower in the group of compliant patients (Table 5).

The potassium and phosphorus values remained consistently within the normal range without showing statistically significant differences (Table 5).

No significant differences were observed in the mean values of the other parameters, specifically albumin, glycemia, hemoglobin, and ferritin, which remained within the expected ranges.

**Figure 6:**
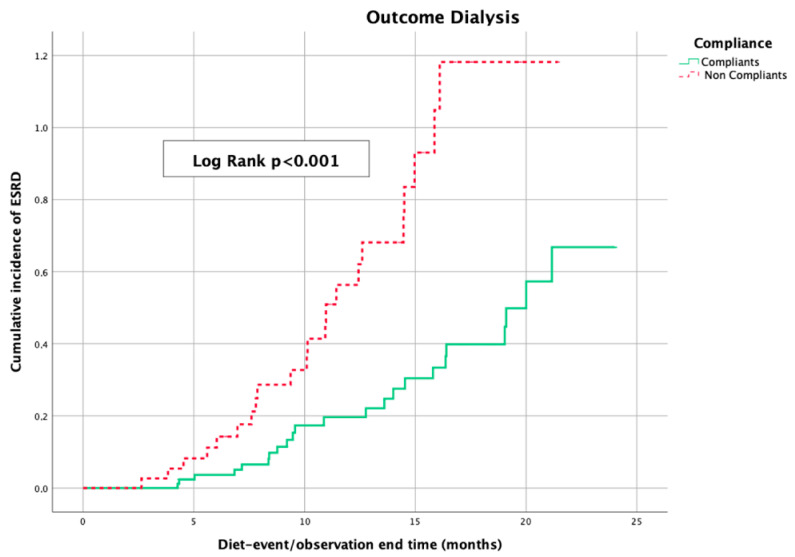
Kaplan–Meier curves showing cumulative incidence of dialysis starting by compliance to sVLPD; ESRD: end-stage renal disease.

The Kaplan–Meier curves, which were used to evaluate the impact of sVLPD on deferring the beginning of dialysis treatment in the two groups of patients, showed a statistical difference (log-rank *p* < 0.001) in favor of the compliant patients (Figure 6).

In the observed population, the Cox regression model confirmed a protective effect of sVLPD regarding the dialysis and for all the other events (renal death), both in the unadjusted analysis and in the analysis adjusted for age, sex, diabetes, heart disease, hypertension, ADPKD, and vascular disease. No influence of sVLPD was registered on mortality (Table 6).

### 3.1. Comparison with eGFR Values before 0.3 g/kg/Protein Diet

In order to verify the actual impact of this DNT to slow down the progression of CKD, we analyzed the eGFR trend in the whole observed population in the year before the onset of sVLPD and in the following two years. We evaluated the differences between patients already on LPD (0.6–0.7–0.8 g/kg/day) and patients not on a diet. A LPD was prescribed to 101 patients (78.3%).

**Figure 7:**
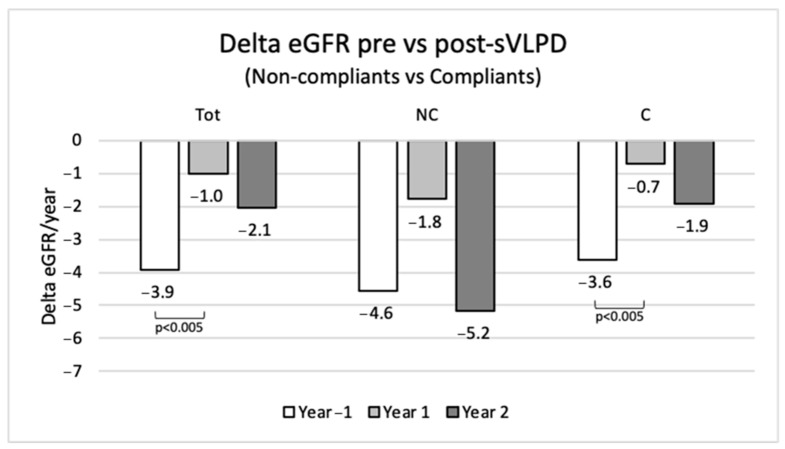
Annual eGFR loss pre- and post-sVLPD in total population and in non-compliant and compliant groups. NC, non-compliants; C, compliants.

In the whole population of the study, the collected data show a mean annual eGFR loss of −3.9 mL/min/1.73 m^2^ in the year before sVLPD, −1 mL/min/1.73 m^2^ in the first year of sVLPD (*p* < 0.005) and −2.1 mL/min/1.73 m^2^ in the second year (Figure 7). Compliant patients in the second year confirmed a slower eGFR reduction compared to non-compliant patients.

**Figure 8:**
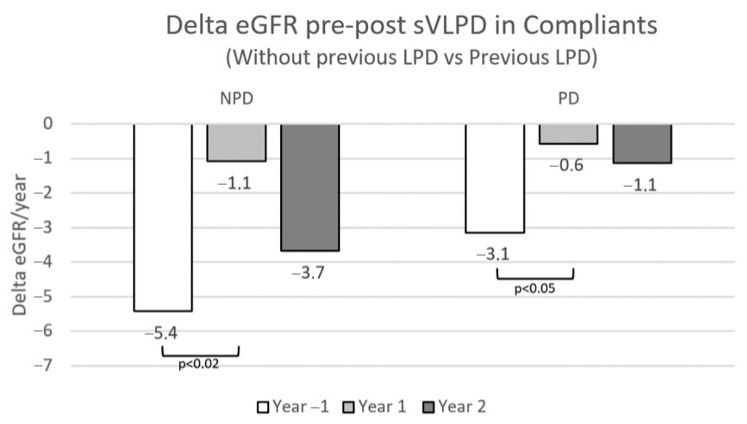
Annual eGFR loss pre- and post-sVLPD in compliant patients with and without previous LPD prescription. NPD, not previous diet; PD, previous diet.

In the sVLPD compliant group, the benefits of this DNT on slowing CKD progression were greatest among the 71 patients who had already been prescribed LPD on CKD stages 3 and 4 (Figure 8).

**Figure 9:**
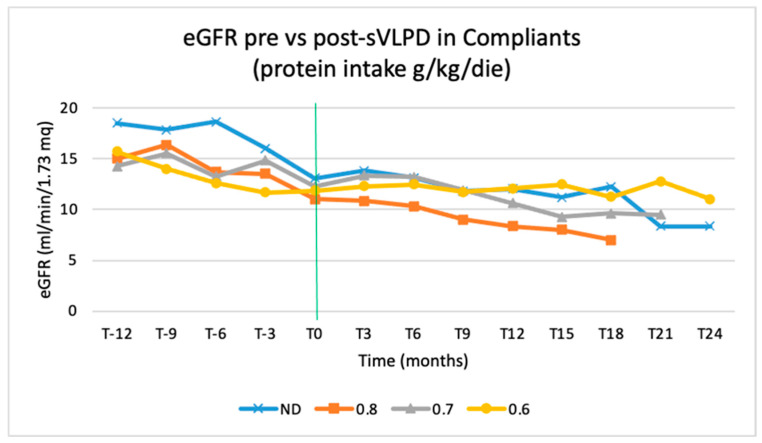
eGFR trends in compliant patients during the 3-year observation by protein intake prescription. ND, not on diet.

The eGFR trend during the sVLPD period is also affected by the type of diet that was previously followed. This trend is inversely proportional to protein intake before the observation period: the trend of patients on a 0.6 g/kg/day diet is better than that of patients on 0.7 and 0.8 g/kg/day diet (Figure 9).

### 3.2. Cost–Benefit Analysis

As already mentioned, the compliance was very high and reached 70.5% in the observed patients (Table 4). Based on the criteria indicated above, 41 patients should have started dialysis treatment already at the beginning of the observation (mean eGFR 8.5 ± 1.4 mL/min/1.73 m^2^) and so we should have had a total of 86 patients during the entire observation period. The actual number of patients that started dialysis treatment was 56 (Table 7); the mean number of avoided months of dialysis was 10.7 ± 6.5/pt for a total of 923.0 months.

The months of dialysis avoided (hemodialysis and peritoneal dialysis) generated gross savings in terms of healthcare expenditure equal to EUR 3,076,470; the expenditure sustained by the Marche region for protein-free products (EUR 90 per patient per month) was EUR 158,358. As a result, the total net savings were EUR 2.918.112 (Table 8).

## 4. Discussion

There are no studies in the literature about patients with CKD stage 5 put on VLPD and supplemented with only essential amino acids, without the use of keto analogues. Furthermore, clinicians are not used to and seem reluctant to adopt systematic and early nutritional therapy in patients with CKD [4].

The analysis from our clinical experience has shown instead that this approach is well tolerated by patients, it is safe in preventing malnutrition and effective in slowing down the progression of CKD in stage 5 patients.

In previous studies based on VLPD supplemented with ketoanalogues [20,26], the reported adherence was poor while in our observation the results show good compliance to DNT.

In particular, a better metabolic control was obtained, and the electrolyte and acid-base balance was maintained. It was possible to control anemia and the iron balance. A slowdown of the progression of the CKD was also observed.

Blood pressure control was better in patients compliant with the diet. This was not evaluated or revealed in previous observations.

The sVLPD that we prescribed is based on protein-free products that replace common starchy foods. The proteins come mainly from vegetable sources. Our diet also includes high quantities of vegetable fats.

The vegetable fats satisfied the daily caloric needs and did not require an increase in the amount of protein-free products, which were gluten-free and would have enhanced the risk of post-prandial hyperglycemia, especially in diabetic patients. It is, therefore, important that all macro nutrients are included in each meal to avoid the glycemic peak.

Furthermore, by prescribing more protein-free products, we would have exceeded the regional dedicated monthly budget and this would have led to higher out-of-pocket expenses for the patients. An adequate amount of vegetables and fruit was also prescribed at each meal.

It should be emphasized that we did not record hyperkalemia in compliant patients despite a predominantly vegetable DNT, and also because patients were given specific indications to avoid foods with a higher content of potassium and phosphorus. The prescribed sodium intake was ≤1.5 g/day (from food alone).

Periodic follow-up by the dietician was fundamental and changes to the diet were made to better adapt it to patients’ individual needs.

The increase in metabolic acidosis, uremic toxins, the inflammatory state and phosphorus all contribute to hypercatabolism in patients with CKD stage 5. As a consequence we were concerned about using sVLPD due to the risk of malnutrition and PEW. It is, therefore, essential to avoid proteins being utilized as energy source by providing an adequate caloric intake. In our observation, malnutrition indices (body composition, albuminemia, metabolic acidosis, transferrin) did not show signs of PEW. This was achieved with careful nutritional follow-ups and patient compliance.

The benefits of ketoanalogues are known but their use negatively affects compliance due to the high number of tablets to be taken. Our data show that it is possible to obtain excellent results using only essential amino acids and to maintain more patients on DNT for longer.

Considering that an improvement in life expectancy and quality of life after starting dialysis treatment has not been demonstrated in elderly patients with pluri-comorbidities, a conservative treatment that reduces uremic symptoms, that slows down the progression of CKD and postpones the replacement therapy, could prevent some patients from developing ESRD.

Our dietary therapeutic solution was adopted as a systematic clinical approach in all patients referred to the advanced renal disease ambulatory, except those with exclusion criteria, and the analysis of data was performed without selection and randomization of patients. This better represents the actual population of stage 5 nephropathic patients that are assisted in nephrology centers.

In our observation, the delay in starting the dialysis treatment appears to be due both to the control of uremic symptoms and to the slowing down of CKD progression, independently of the underlying nephropathy and comorbidities.

Our opinion is that the patients should follow the dietary nutritional treatment as long as possible, up to the onset of events or eventual signs of PEW. Further studies should be conducted to evaluate the long-term safety of sVLPD also to establish whether the effects on CKD progression using an LPD diet (0.6 g/kg/die + essential amino acids) are equal in efficacy to an sVLPD with amino acids.

The possibility of implementing incremental dialysis programs when DNT alone is no longer sufficient in the clinical control of uremia should not be overlooked.

Our analysis has methodological limitations because it is a non-interventional longitudinal observational study based on real clinical experience without the design of a randomized and/or controlled study. We did not take into account the effects on proteinuria, on the dosage of drugs (e.g., erythropoietins, iron, P and K chelators, antihypertensives, bicarbonate, antidiabetics), on glycemic and lipid control, or on the ultrasound evaluation of the muscle and strength. These will be addressed in a future study.

There is already known evidence that demonstrates how a highly hypoproteic diet brings savings for the healthcare system, reducing not only the costs associated with the dialysis treatment but also those related to hospitalization, the management of vascular and peritoneal accesses, and the management of clinical complications (Scalone DNT 2010). The slowdown in the progression of the CKD generated important economic savings in terms of “time without dialysis”, considering that in the calculation model we used underestimated criteria compared to “real life” (cut-off value of eGFR for starting dialysis in a patient not on diet = 10 mL/min/1.73 m^2^, average cost of dialysis treatment EUR 40,000/pc/year, estimated average loss of eGFR 3 mL/min/year).

The benefits in terms of a reduction in water consumption (about 500 L/seat HD/pt) and waste production (about 2 kg/seat HD/pt) should also be considered. During our experience, we were able to save around 5.5 million liters of water and about 22 tons of waste.

## 5. Conclusions

Our observational study and our clinical experience suggest that a new approach of diet therapy is achievable and safe and that malnutrition can be avoided. Specifically, it is effective in slowing down the progression of chronic kidney disease and postponing the replacement dialysis treatment, with consequent economic savings for the NHS. Furthermore, it prolongs the predialytic time, allowing patients to choose the most appropriate dialysis treatment and to achieve the goal of pre-emptive transplantation.

The DNT that we adopted proved to be beneficial in the treatment of CKD stage 5 patients because of the high compliance obtained by making supplementation easier to follow. We only prescribed essential amino acids, reducing the overall tablet intake. According to our experience, the main requirements are a personalized diet and close nephrological and nutritional follow-up.

Another interesting observation from the data analyzed is that the benefits would be greater if low-protein diets were prescribed from the early stages of CKD.

Further studies should are needed to confirm our analysis in the CKD stage 5 patients population. It would be interesting to explore the effectiveness of sVLPD in patients at other CKD stages of the disease in future studies.

## Figures and Tables

**Table 1:** Characteristics of the population under observation.

Variables	N	Mean ± SD/%
Tot pts	129	
Age		75.2 ± 13.7
Sex (M/F)	78/51	60.5/39.5
Comorbidity		
Hypertension	129	100
Diabetes	52	40.3
Cardiopathy	32	24.8
ADPKD	16	12.4
Vasculopathy	53	41.1
Previous diet		
None	28	21.7
0.6 (g/kg/die)	58	45.7
0.7 (g/kg/die)	29	21.0
0.8 (g/kg/die)	14	12.4
Tot in LPD	101	78.3

Abbreviations: SD, standard deviation; ADPKD, autosomic dominant polycystic kidney disease; LPD, low-protein diet.

**Table 2:** Composition and dosage of amino acids used.

Amino Acids	Daily Dose—2 Soluble Sachets (mg)
L-Leucine	2500
L-Lysine	1300
L-Isoleucine	1250
L-Valine	1250
L-Threonine	700
L-Cystine	300
L-Histidine	300
L-Phenylalanine	200
L-Methionine	100
L-Tyrosine	60
L-Tryptophan	40
Total	8000

**Table 3:** Parameter values at baseline, in the general population and in the two subgroups, compliants and non-compliants.

Parameter	Tot Population (Mean/Median ± SD/IQR)	Compliants (Mean/Median ± SD/IQR)	Not Compliants (Mean/Median ± SD/IQR)	Sig (*p* < 0.05)
Age (years)	75.2 ± 13.7	76.5 ± 13.5	72.2 ± 13.5	0.1
Weight (kg)	75.7 ± 16.2	71.9 ± 12.8	84.2 ± 19.1	<0.002
BMI (kg/m^2^)	27.7 ± 5.6	26.8 ± 4.5	29.5 ± 7	<0.05
SBP (mmHg)	149.4 ± 20.5	149.2 ± 19.2	151.3 ± 19.5	0.62
DBP (mmHg)	78.4 ± 10.3	78.2 ± 10.6	79 ± 9.1	0.71
Urea (mg/dL)	140.9 ± 39.8	136.9 ± 34.8	150.3 ± 48	0.13
Creatinine (mg/dL)	4.5 ± 1.3	4.3 ± 1.2	4.8 ± 1.3	<0.05
eGFR (mL/min/1.73 m^2^)	12.1 ± 3.2	12.1 ± 3.1	11.9 ± 3.4	0.75
Uric acid (mg/dL)	5.5 ± 2.1	5.5 ± 2.3	5.5 ± 1.6	0.98
Glycaemia (mg/dL)	108.3 ± 36.7	107.6 ± 39	109.9 ± 29.3	0.75
Ca (mg/dL)	9.4 ± 0.8	9.3 ± 0.9	9.5 ± 0.6	0.17
P (mg/dL)	4.4 ± 0.8	4.4 ± 0.8	4.3 ± 0.7	0.76
K (mEq/L)	4.6 ± 0.6	4.5 ± 0.6	4.6 ± 0.6	0.45
Na (mEq/L)	140.3 ± 4.8	140.3 ± 5.4	140.3 ± 2.8	0.94
Cl (mEq/L)	105 ± 4.6	104.9 ± 4.4	105 ± 4.9	0.95
Mg (mg/dL)	2.2 ± 0.3	2.2 ± 0.3	2.3 ± 0.4	0.30
Albumin (g/dL)	3.9 ± 0.4	3.9 ± 0.3	3.9 ± 0.5	0.83
Haemoglobin (g/dL)	11.3 ± 1.4	11.2 ± 1.3	11.4 ± 1.6	0.45
Ferritin (ng/mL)	92.4 (62.7–186.7)	102.3 (68.5–183.1)	84.1 (47.8–206)	0.71
Sidaeremia (mcg/dL)	61.8 ± 24.4	61.8 ± 25.1	61.7 ± 21.8	0.99
Trasferrin (mg/dL)	191.5 ± 35.3	189.5 ± 36.7	195.8 ± 28.3	0.85
TSAT (%)	21.9 ± 11.5	22.1 ± 12.4	21.1 ± 8	0.70
pH	7.3 ± 0.1	7.32 ± 0.08	7.35 ± 0.07	0.28
Bicarbonates (mmol/L)	22.8 ± 2.8	22.6 ± 2.6	23.6 ± 2.8	0.45
Protein intake (g/kg/die)	0.87 ± 0.3	0.78 ± 0.27	1.01 ± 0.31	0.08

Abbreviations: SD, standard deviation; IQR, Interquartile range; BMI, body mass index; SBP, systolic blood pressure; DBP, diastolic blood pressure; TSAT, transferrin saturation.

**Table 4:** Summary of the observation in relation to events, diet duration and compliance.

	N	Mean ± SD/%
Events	Tot 99	
Death	24	18.6
Transplantation	4	3.1
HD	36	27.9
PD	20	14.0
Recovery	15	11.6
Mean time from diet start to event/observation end (months). Total months on diet	1759.5	13.6 ± 6.8
Compliance		
Compliant pts	91	70.5
Non-compliant pts	38	29.5

Abbreviations: SD, standard deviation; HD, hemodialysis; PD, peritoneal dialysis.

**Table 5:** Comparison of the mean values of the parameters monitored in the two groups during the observation.

Parameters	Compliants (Mean/Median ± SD/IQR)	Non-Compliants (Mean/Median ± SD/IQR)	Sig (*p* < 0.05)
Weight (kg)	70.8 ± 12.3	97.1 ± 20.8	<0.002
BMI (kg/m^2^)	26.2 ± 4	31.8. ± 7.4	<0.005
SBP (mmHg)	136.9 ± 11.8	150.1 ± 7	<0.01
DBP (mmHg)	75 ± 3.1	79.4 ± 1	<0.001
Urea (mg/dL)	116.1 ± 31.7	174.2 ± 42.2	<0.002
Creatinine (mg/dL)	4.6 ± 0.3	6.1 ± 1.2	<0.001
eGFR (mL/min/1.73 m^2^)	11.6 ± 3.3	9.3 ± 2.7	<0.05
Uric acid (mg/dL)	4.9 ± 0.6	5.6 ± 0.5	<0.01
Glycaemia (mg/dL)	104.9 ± 6.4	107.8 ± 7.6	0.29
Ca (mg/dL)	9.5 ± 0.6	9.2 ± 0.6	0.12
P (mg/dL)	4.3 ± 0.9	4.9 ± 1.0	0.15
K (mEq/L)	4.5 ± 0.5	4.5 ± 0.5	0.35
Na (mEq/L)	139. ± 2.7	139.6 ± 4.0	0.43
Cl (mEq/L)	103.4 ± 4.4	104 ± 3.3	0.33
Mg (mg/dL)	2.2 ± 0.4	2.3 ± 0.3	0.07
Albumin (g/dL)	3.9 ± 0.4	4.0 ± 0.4	0.06
Haemoglobin (g/dL)	11.2 ± 1.1	11.4 ± 1.4	0.95
Ferritin (ng/mL)	187.7 (145.6–202)	145.9 (97–158)	0.06
Sidaeremia (mcg/dL)	60.3 ± 20.3	59.5 ± 18.7	0.59
Trasferrin (mg/dL)	183.9 ± 33.8	200.1 ± 25.4	<0.02
TSAT (%)	24 ± 9.0	21.7 ± 6.9	<0.005
pH	7.34 ± 0.1	7.35 ± 0.06	0.53
Bicarbonates (mmol/L)	24.2 ± 1.9	23.9 ± 2.5	0.98
Protein intake (g/kg/die)	0.55 ± 0.1	0.87 ± 0.55	<0.01
Body water (% pts in range)	18.1 ± 12.0	15.2 ± 14.2	0.64
Lean mass (% pt in range)	59.3 ± 12.6	51.1 ± 33.9	0.54
Fat mass (% pt in range)	75.0 ± 14.0	54.0 ± 36.1	0.18

Abbreviations: SD, standard deviation; IQR, interquartile range; BMI, body mass index; SBP, systolic blood pressure; DBP, diastolic blood pressure; TSAT, transferrin saturation.

**Table 6:** Cox regression model for sVLPD on main outcomes with compliance as discriminating factor.

Characteristics	Non-Adjusted HR (95% CI, Sig.)	Adjusted (95% CI, Sig.)
Dialysis	0.377 (0.223–0.636, *p* < 0.001)	0.361 (0.200–0.650), *p* = 0.001)
All the events/renal death	0.513 (0.328–0.805, *p* < 0.005)	0.465 (0.278–0.777, *p* < 0.005)
Death	1.285 (0.473–3.487, *p* = 0.790)	0.969 (0.302–3.110, *p* = 0.636) *

* adjusted HR significative for age (*p* < 0.001). Abbreviations: HR, hazard ratio; CI, confidence interval.

**Table 7:** Breakdown of patients starting on dialysis treatment and months of dialysis avoided.

Parameters	N	Media ± SD/%
Pts should start dialysis at the beginning of the observation (eGFR ≤ 10 mL/min/1.73 m^2^)	41	
Pts estimated to start dialysis during observation	86	
Pts started dialysis (HD + PD)	56	43.5
Dialysis months avoided (tot)	923.0	
Dialysis months avoided/pt		10.7 ± 6.5

Abbreviations: SD, standard deviation; HD, hemodialysis; PD, peritoneal dialysis.

**Table 8:** Report of costs and savings deriving from the prescription of sVLPD.

	Euro
Cost–benefit analysis	
Total saving for dialysis	3.076.470
Diet total cost	158.358
Total gross saving	2.918.112
Net saving/month	3.161
Net total saving/pt	33.932

## Data Availability

The data presented in this article are available on request from the corresponding author.

## Supplementary Materials

The following supporting information can be downloaded at: https://www.mdpi.com/article/10.3390/nu15163568/s1, Figure S1: Percentage of patients on target for lean and fat mass during the observational period; Figure S2: Mean values of pH and bicarbonates during observational period in Non-compliants (NC) vs. Compliants (C).
