# Supplementary material for: Supplemented Very Low Protein Diet (sVLPD) in Patients with Advanced Chronic Renal Failure: Clinical and Economic Benefits
Source: Nutrients. 2023 Aug 13;15(16):3568. doi: 10.3390/nu15163568 (PMC10457928; doi:10.3390/nu15163568)
Supplement: Supplementary file 1 [file nutrients-15-03568-s001.zip › nutrients-2521192-supplementary.pdf]

nutrients MDPI

Supplementary material

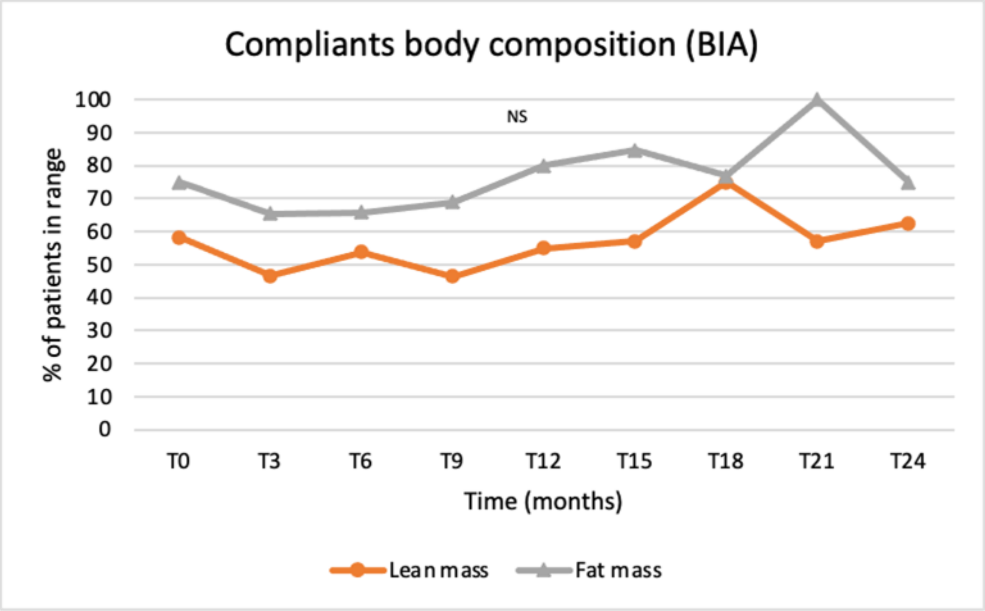

Supplement Figure S1. Percentage of patients on target for lean and fat mass during the observational period

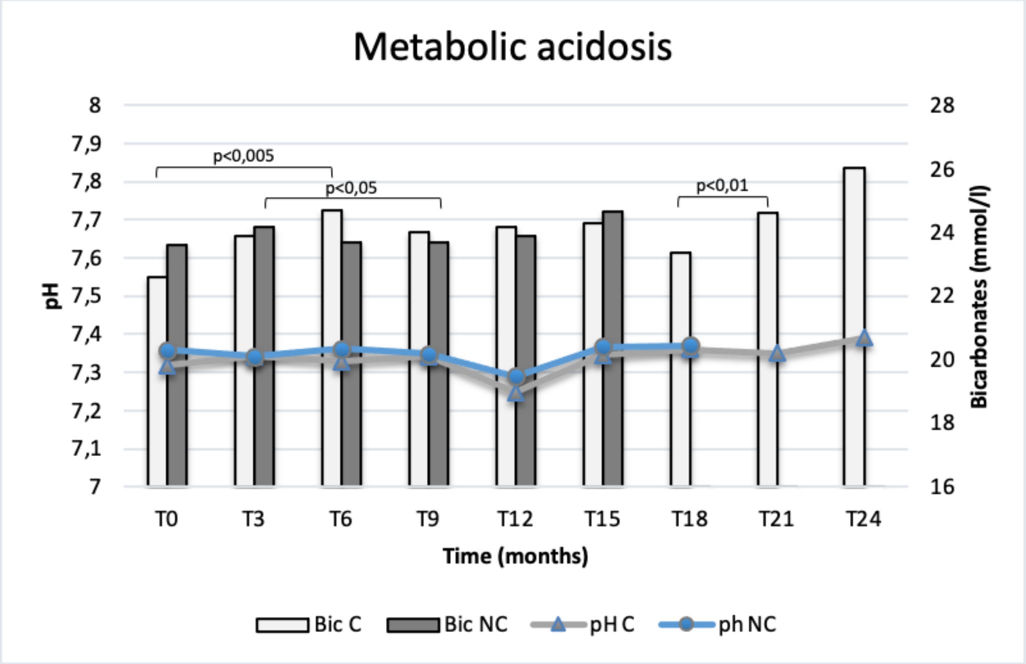

Supplement Figure S2. Mean values of pH and bicarbonates during observational period in Non-compliants (NC) vs Compliants (C).
